# Management of Giant Splenic Artery Aneurysm: Comprehensive Literature Review

**DOI:** 10.1097/MD.0000000000001016

**Published:** 2015-07-13

**Authors:** Sami Akbulut, Emrah Otan

**Affiliations:** Department of Surgery and Liver Transplant Institute, Inonu University Faculty of Medicine, Malatya, Turkey.

## Abstract

To provide an overview of the medical literature on giant splenic artery aneurysm (SAA).

The PubMed, Medline, Google Scholar, and Google databases were searched using keywords to identify articles related to SAA. Keywords used were splenic artery aneurysm, giant splenic artery aneuryms, huge splenic artery aneurysm, splenic artery aneurysm rupture, and visceral artery aneurysm. SAAs with a diameter ≥5 cm are considered as giant and included in this study. The language of the publication was not a limitation criterion, and publications dated before January 15, 2015 were considered.

The literature review included 69 papers (62 fulltext, 6 abstract, 1 nonavailable) on giant SAA. A sum of 78 patients (50 males, 28 females) involved in the study with an age range of 27–87 years (mean ± SD: 55.8 ± 14.0 years). Age range for male was 30–87 (mean ± SD: 57.5 ± 12.0 years) and for female was 27–84 (mean ± SD: 52.7 ± 16.6 years). Most frequent predisposing factors were acute or chronic pancreatitis, atherosclerosis, hypertension, and cirrhosis. Aneurysm dimensions were obtained for 77 patients with a range of 50–300 mm (mean ± SD: 97.1 ± 46.0 mm). Aneurysm dimension range for females was 50–210 mm (mean ± SD: 97.5 ± 40.2 mm) and for males was 50–300 mm (mean ± SD: 96.9 ± 48.9 mm). Intraperitoneal/retroperitoneal rupture was present in 15, among which with a lesion dimension range of 50–180 mm (mean ± SD; 100 ± 49.3 mm) which was range of 50–300 mm (mean ± SD: 96.3 ± 45.2 mm) in cases without rupture. Mortality for rupture patients was 33.3%. Other frequent complications were gastrosplenic fistula (n = 3), colosplenic fistula (n = 1), pancreatic fistula (n = 1), splenic arteriovenous fistula (n = 3), and portosplenic fistula (n = 1). Eight of the patients died in early postoperative period while 67 survived. Survival status of the remaining 3 patients is unclear. Range of follow-up period for the surviving patients varies from 3 weeks to 42 months.

Either rupture or fistulization into hollow organs risk increase in compliance with aneurysm diameter. Mortality is significantly high in rupture cases. Patients with an evident risk should undergo either surgical or interventional radiological treatment without delay.

## INTRODUCTION

Splenic artery aneurysms (SAAs) account for more than half of all visceral artery aneurysms.^1^ SAAs are the third most frequent intraabdominal aneurysms, following abdominal aorta and iliac artery aneurysms. Histopathologically, SAAs are classified into 2 types: true and pseudoaneurysms. Despite being rare, SAAs are important due to their potentially life-threatening complications, such as spontaneous intraperitoneal rupture, rupture into the neighboring hollow organs, and fistulization into the pancreatic duct.^1,2^ Most small SAAs (≤ 2 cm) are asymptomatic, and are diagnosed incidentally when radiological tests are performed for another condition.^3^ In contrast, most giant SAAs (≥5 cm) are symptomatic and can result in complications. The most frequent management options for SAAs are medical treatment, close follow-up, open surgery, endovascular treatment, and laparoscopic surgery.^1,4–9^ The aim of this review was to provide an overview of the medical literature on giant splenic artery aneurysm (SAA).

## METHODS

**FIGURE 1:**
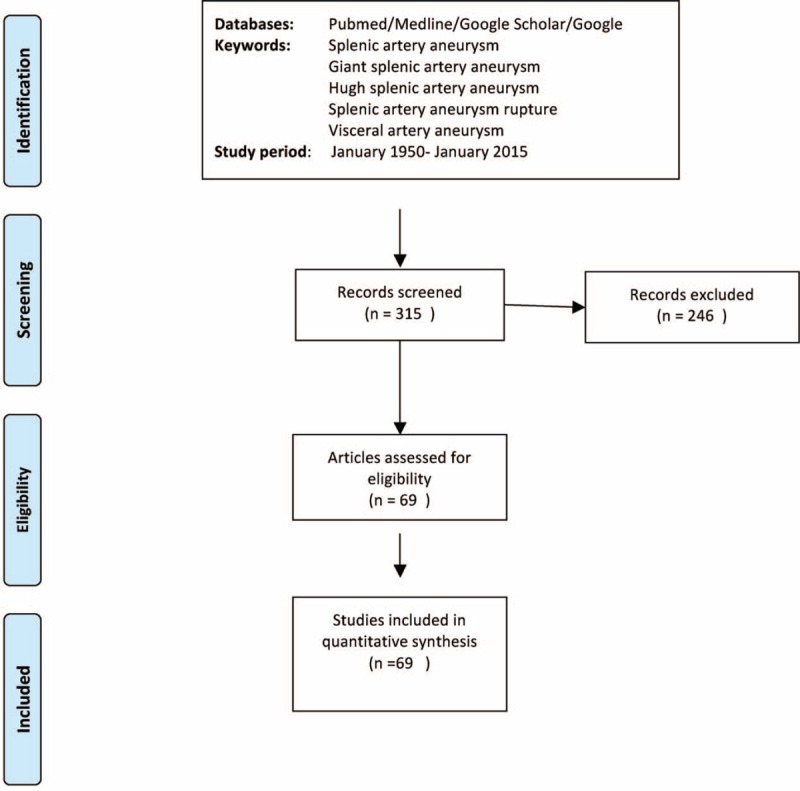
Flow diagram: patient's selection and exclusion criteria.

The main objective of this study was to evaluate the medical literature to identify studies on giant splenic artery aneurysm published from January 1950 to January 2015. To achieve this purpose, we scanned the PubMed, Medline, Google Scholar, and Google databases for the keywords “splenic artery aneurysm,” “giant splenic artery aneurysm,” “huge splenic artery aneurysm,” “splenic artery aneurysm rupture,” and “visceral artery aneurysm” entered alone or in various combinations (Figure 1flow diagram). The language of the publication was not a limitation criterion. Despite the lack of a consensus definition on the dimensions of a giant aneurysm, most of the studies defined a giant aneurysm as being bigger than 5 cm^5^; therefore, aneurysms of that size were included in this study. All of the case reports, letters to editors, review articles, and original studies of SAAs were reviewed, and their reference lists were evaluated. The corresponding authors of studies with missing data that hindered comparisons were contacted via email and asked for the required data. When we were unable to contact the corresponding authors, the editorial offices were contacted via email. Studies without the full text, an abstract with insufficient data, or studies with poor content for comparison were excluded. Some of the tables in the review studies were also useful. All of the studies were evaluated by Dr Akbulut. A table was constructed for the studies that included publication year, country, article language, article type (e.g., case report), text type (full text/abstract), patient age, sex, aneurysm size (mm), possible etiological factors, aneurysm type (true/pseudo), presentation data (symptomatic/incidental), examination findings, radiological tools, preoperative complications (clinical or radiological), management (radiological intervention, surgery, conservative treatment, or combined), outcome, and follow-up. One goal of this literature review was to clarify this terminology. The ultrasonographic (US), computed tomographic (CT), and angiographic dimension calculations for the same lesion differed in some of the studies. Therefore, the most suitable dimensions were chosen and included in the table. In addition, there was contradictory information about the diagnostic procedures used for some of the lesions, and whether they were found incidentally or were symptomatic. This led us to construct an algorithm that classified aneurysms as incidental (asymptomatic) when coincidentally diagnosed during radiological interventions for another condition, or as symptomatic when they resulted in complaints requiring medical help. The symptoms ranged from abdominal pain, a feeling of fullness, and a palpable mass to more serious conditions, such as gastrointestinal bleeding, perforation and gastrointestinal fistula formation, and portal hypertension due to compression by surrounding organs. It was difficult to determine whether the symptoms arose from pancreatitis or the aneurysm itself in cases accompanied by acute or chronic pancreatitis. The same is true for pancreatic pseudocysts. We classified all such cases as symptomatic SAA. Due to retrospective design of this literature review, we did not apply for ethics committee approval. The patient ages and aneurysm dimensions are given as the mean ± standard deviation (SD) and range.

## RESULTS

The literature review included 69 papers involving 78 patients with giant SAAs (≥5 cm) that met the above-mentioned criteria.^1–8,10–68,71,72^ The mean ± SD age was 55.8 ± 14.0 (range 27–87) years: 28 women [age 52.7 ± 16.6 (range 27–84) years] and 50 males [age 57.5 ± 12.0 (range 30–87) years]. The male:female ratio was 1.79:1. Of the included cases, aneurysm dimensions were obtained for 77 patients, and the mean was 97.1 ± 46.0 mm (range 50–300 mm):97.5 ± 40.2 mm (range 50–210 mm) in women and 96.9 ± 48.9 mm (range 50–300 mm) in men. Of the 69 papers, 61 were published in English, 6 were in Turkish, and 1 each was in French and Italian. Forty-five articles were case reports, 9 were case reports with a literature review, 3 were case series, 3 were clinical images, 2 were original papers, 2 were poster presentations, 2 were letter to the editor, 1 was clinical imaging with a literature review, and the paper type of remaining 2 articles were nonavailable. Of the articles, 14 originated in the United States, 9 each originated in Turkey and India, 7 in Italy, 6 in China, 4 in the United Kingdom, 3 in Japan, 2 each in Taiwan, Greece, Poland, and Canada, and 1 each from Brazil, Bahrain, Belgium, Morocco, Serbia, Tunisia, Spain, Pakistan, and France. Full text was obtained for 62 of the 69 papers, whereas only abstracts were available for 6 papers, and no text of any kind was available for 1 paper. For the studies in which the entire text was not available, study details were obtained from either abstracts or a literature review from the study.^28,50,57,59,64–66^ In addition, some important information was given in the table footnotes. The remaining clinical and demographic details are given in Table 1     . The necessary information is also given in the relevant section of discussion.

**TABLE 1:**
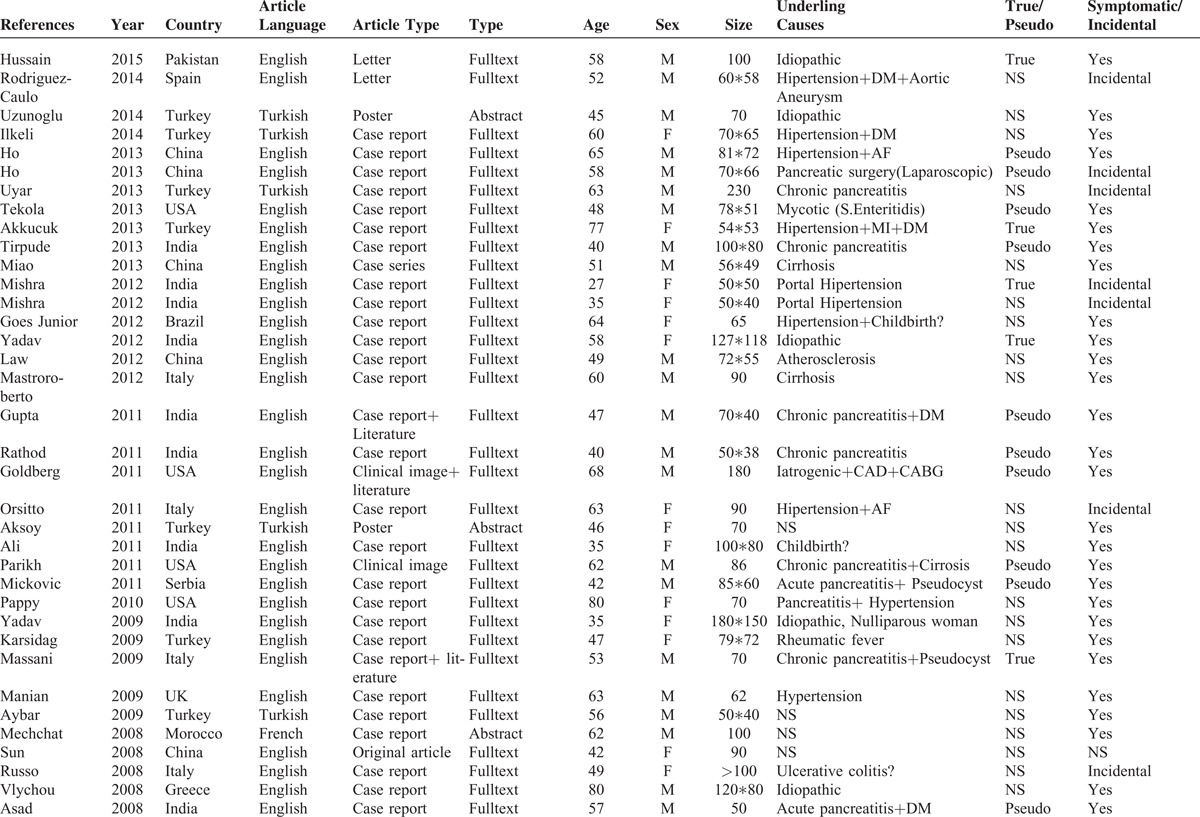
Demographic and Clinical Characteristics of 78 Patients With Giant SAAs

**TABLE 1 (Continued):**
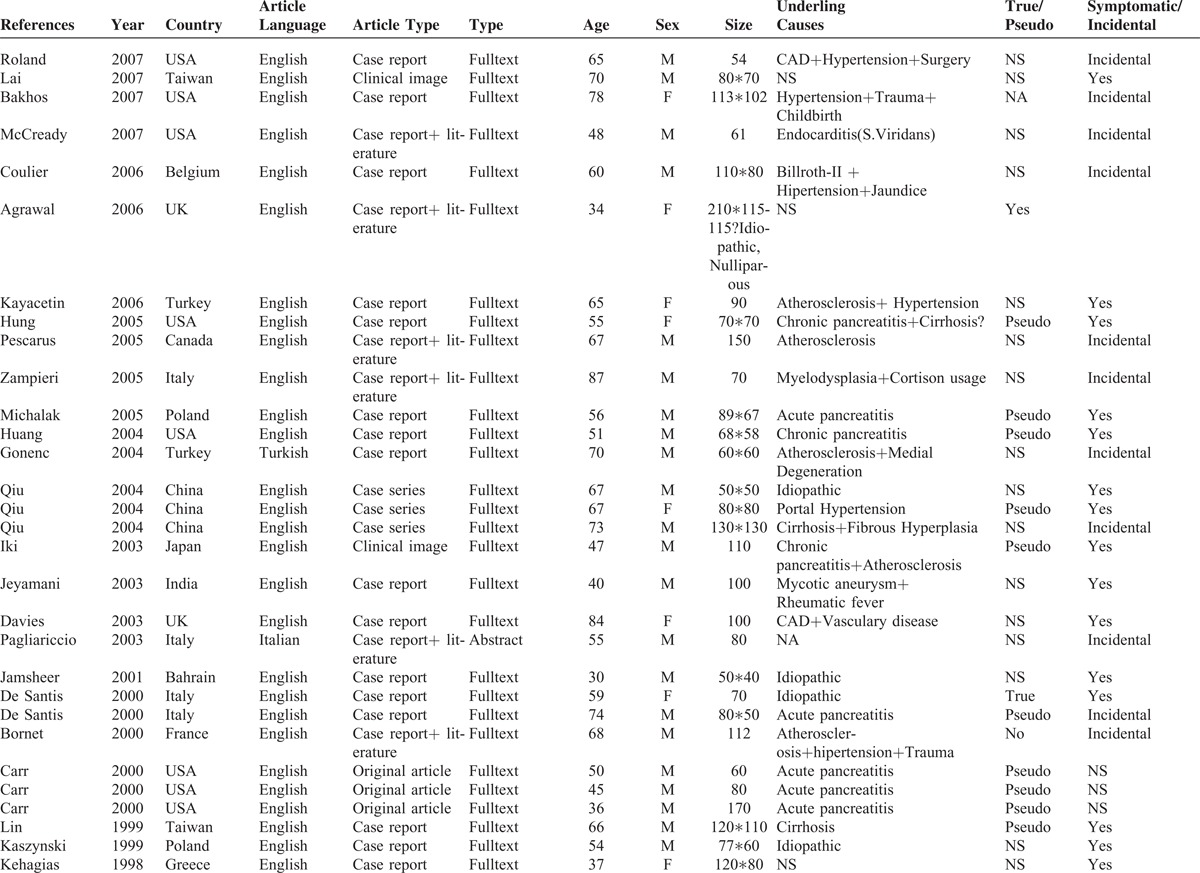
Demographic and Clinical Characteristics of 78 Patients With Giant SAAs

**TABLE 1 (Continued):**
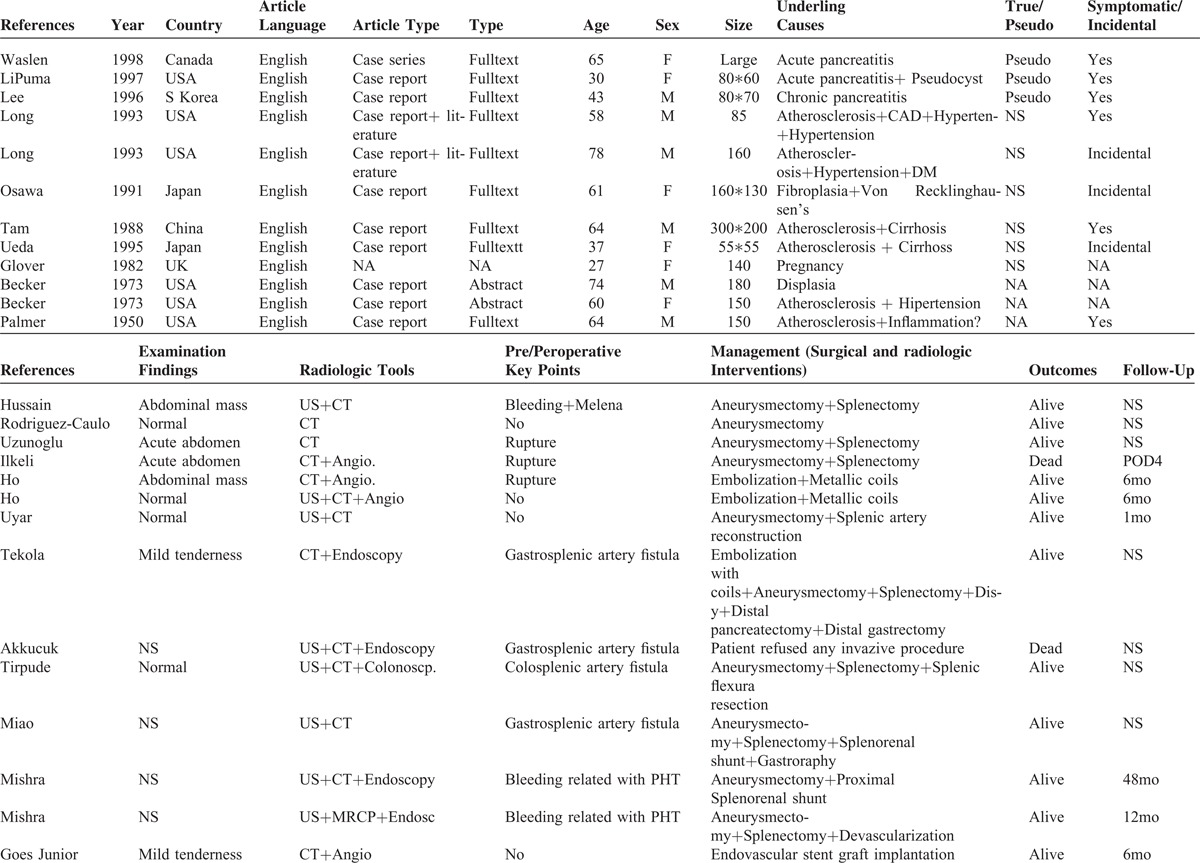
Demographic and Clinical Characteristics of 78 Patients With Giant SAAs

**TABLE 1 (Continued):**
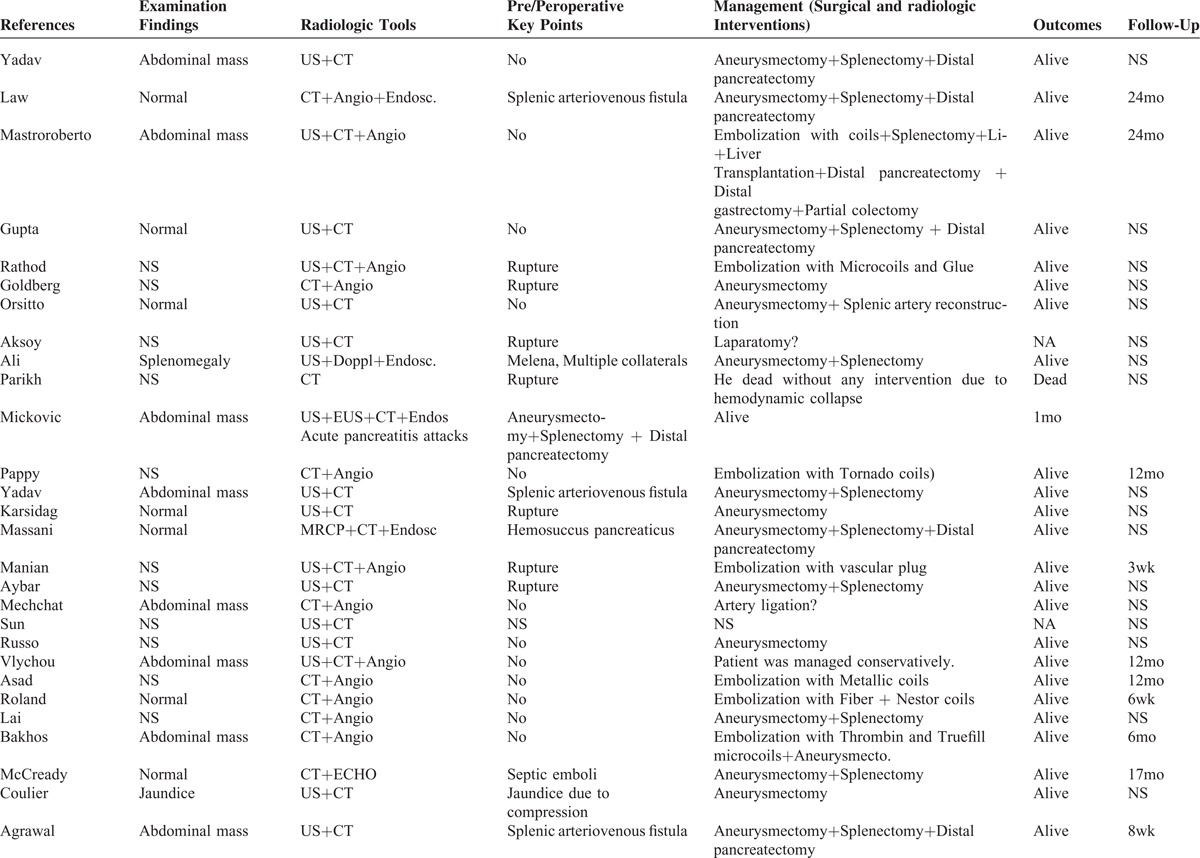
Demographic and Clinical Characteristics of 78 Patients With Giant SAAs

**TABLE 1 (Continued):**
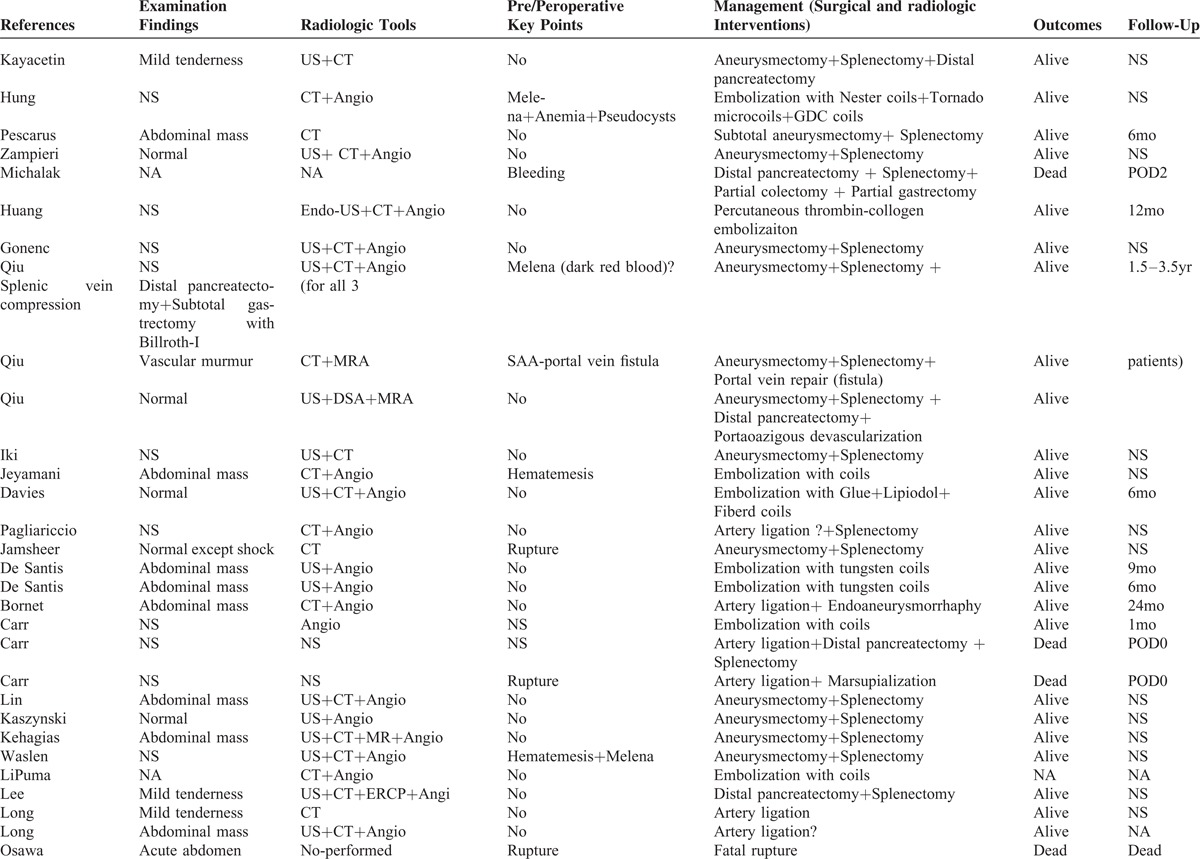
Demographic and Clinical Characteristics of 78 Patients With Giant SAAs

**TABLE 1 (Continued):**
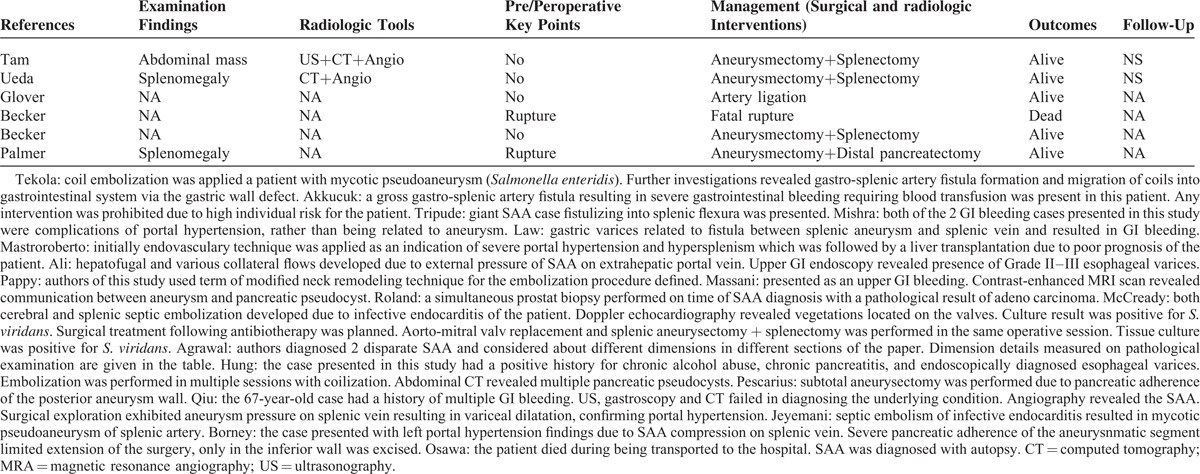
Demographic and Clinical Characteristics of 78 Patients With Giant SAAs

## DISCUSSION

### Epidemiologic Features

Arterial aneurysms, defined as an artery with a diameter of 50% bigger than expected, constitute the vast majority of vascular diseases, and result in mortality and morbidity.^11,16^ These aneurysms are classified as fusiform or saccular based on their morphology and dimension. Splanchnic aneurysms comprise 5% of intraabdominal aneurysms, and involve the celiac and superior and inferior mesenteric arteries and their branches. SAAs are the most frequent splanchnic artery aneurysms, constituting 50–70% of these aneurysms.^7,9,15^ In addition, SAAs are the third most frequent intraabdominal aneurysms, following abdominal aorta and iliac artery aneurysms.^13,17^ SAAs were first defined by Beaussier in 1770 in a cadaver study.^15,17,69^ Hoegler diagnosed these lesions preoperatively with radiologic interventions in 1920.^69^ Although the true incidence of SAA is unclear, various studies of autopsies, angiography findings, and autopsies of individuals ≥60 years revealed rates of 0.01–0.2%, 0.78–0.80%, and 10.4%, respectively.^1,7,17,18,28,69,70^ SAAs are 4 times more frequent in women, and are most commonly diagnosed in people 52–61 years of age.^1,7,9,11,22^ Our literature survey revealed that giant SAAs are 1.78 times more frequent in males, and the mean age at diagnosis is 57.5 years for males and 52.7 for females. These results suggest that SAAs are diagnosed at an older age in male patients when compared to female patients.

### Pathology

Although the pathogenesis of SAA remains unclear, loss of the media layer characterized by disintegration of elastic fibers and loss of smooth muscles is the most frequent finding.^1,7,16^ Histopathologically, SAAs are classified into 2 types: true and pseudoaneurysms.^16^ True aneurysms are vascular enlargements involving all 3 layers of the artery wall: the intima, media, and adventitia. Pseudoaneurysms are enlargements that do not contain 1 or 2 layers of the artery wall.^16^ Calcification, intimal hyperplasia, arterial dysplasia, fibromuscular dysplasia, and medial degeneration are the most frequent histopathological findings. Most SAAs arise from the main body of the splenic artery. Of the true SAAs, 74–87%, 20–22%, and <6% originate from the distal third, middle, and proximal thirds, respectively.^25,26^ Most mycotic aneurysms arise from the splenic artery bifurcation. Nearly 75% of SAAs are solitary and saccular.^11,13,16^ At the time of diagnosis, the mean diameter is 2.1 cm, and rarely exceeds 3 cm.^7,26,69^ Although some authors define aneurysms greater than 10 cm in diameter as giant lesions, most use 5 cm as the threshold, which was also the threshold used in this study.^1,5,12,16,19,22,26^

### Etiology and Possible Risk Factors

Absolute risk factors for SAAs are not known, and possible risk factors for true and pseudoaneurysms vary.^16^ True SAAs are more frequent than pseudo SAAs; the main risk factors for true SAAs are hypertension, atherosclerosis (possibly due to hypertension), cirrhosis, portal hypertension (PHT), liver transplantation, female sex, pregnancy, and multiparity.^7,13,14,22,70^ Pregnancy and PHT are the most important factors. The incidence of SAA among patients with a diagnosis of cirrhosis and portal hypertension varies from 7 to 50%, and PHT is diagnosed in 50% of SAA patients.^3,18,33^ Hormonal changes during pregnancy and increased splenic artery outflow might contribute to either the improvement of a new aneurysm, enlargement of a preexisting lesion, or rupture of an aneurysm.^7,14,18^ The third trimester of pregnancy is the period with the highest risk.^1^ Our literature survey revealed that among the 78 giant SAA cases, 8 had cirrhosis,^13,18,23,41,47,56,64,65^ 2 had portal hypertension,^14^ and 1 had a history of pregnancy,^66^ which are possible predisposing conditions for SAA. Relatively rare risk factors are splenomegaly, medial fibrodysplasia, arteritis, collagen vascular disease, polyarteritis nodosa, systemic lupus erythematosus, anomalous splenic artery origin, α_1_-antitrypsin deficiency, and inflammatory and infectious diseases.^8,14^ Predisposing conditions for pseudo-SAAs are pancreatitis (chronic or acute), pancreatic pseudocyst, and abdominal trauma.^13,19,20^ Pancreatitis is the main risk factor for pseudo-SAA. Pancreatic enzymes disintegrate elastin fibers as a result of arterial wall destruction, which predisposes one to pseudoaneurysm development.^12,16,20^ Similarly, the development of a cysto-aneurysmal fistula as a result of arterial wall erosion by the cyst wall is a well-known condition with pancreatic pseudocysts.^19,24,34^ Abdominal and endovascular surgery, infective endocarditis, and peptic ulcer disease are relatively rare risk factors.^13,19^ Pancreatitis might be the most remarkable condition, considering its overall incidence.^70^ Our literature survey found that the predisposing condition in 25.6% of the 78 giant SAA cases was pancreatitis. The most common possible risk factor for SAA is summarized in Table 2.

**TABLE 2:**
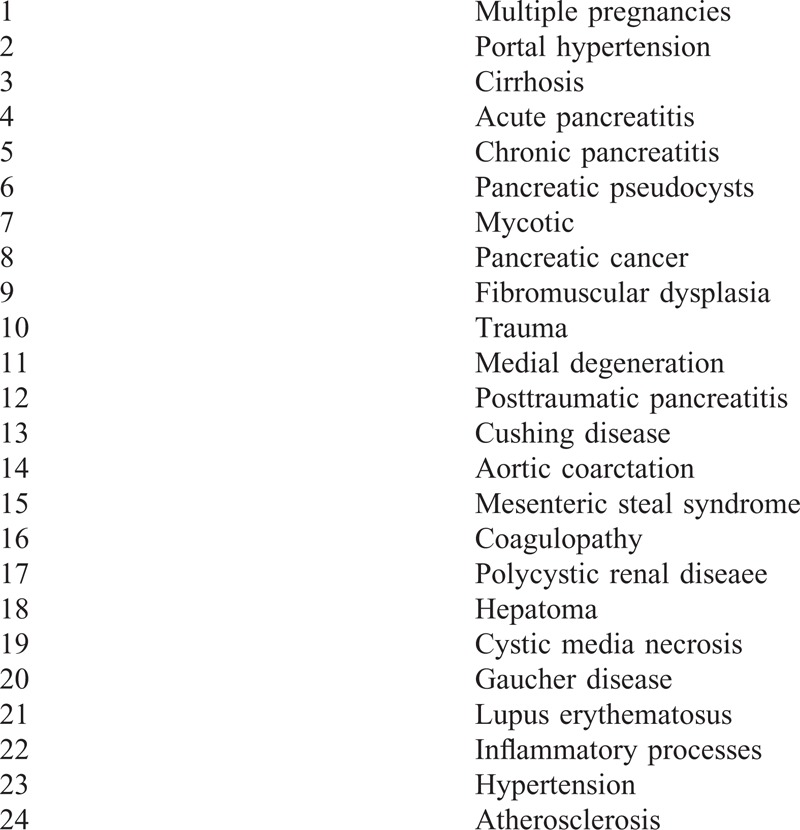
The Possible Causes Are Related to the Development of Splenic Artery Aneurysms

### Clinical Aspects

Among SAA cases, 80% are asymptomatic and diagnosed incidentally during a radiological intervention for another cause.^13,22,69^ The most frequent symptoms for symptomatic cases are epigastric and left-upper quadrant abdominal pain. Some cases might also present with a general gastrointestinal complaint of a feeling of fullness, loss of appetite, nausea, or vomiting.^8,13^ Aneurysms with relatively bigger dimensions might be detected as a pulsatile mass in the upper-left quadrant or epigastrium. We believe that studies defining the proportion of SAAs that are symptomatic are not accurate. Nearly all studies give incidence rates citing another study. In addition, few have examined the relationship between aneurysm dimensions and rates in detail. For example, for 78 giant SAA cases with diameters ≥5 cm, presentation data were obtained for 71 cases, and we determined that 70.4% of them were symptomatic and 29.6% were diagnosed incidentally. The difference between our finding and other studies reveals that the dimensions of aneurysms are related to symptoms. However, the heterogeneity of the symptoms considered in those studies limits our ability to draw conclusions. Symptoms might arise from either the SAA or the underlying disease (e.g., pancreatitis).

Spontaneous SAA rupture, fistulization into neighboring organs (stomach, duodenum, colon), a splenic arteriovenous fistula between the SAA and portal vein, retroperitoneal rupture, and fistulization into the duct of *Wirsun*g are the most frequent and life-threatening complications of SAAs, of which spontaneous rupture is the most dangerous.^1,69^ The risk of spontaneous rupture of a SAA is 2–10%, and the mortality rate following rupture is 10–40%.^1,11,13,16,26,69^ In comparison, the rupture risk for giant SAAs increases to 28%, with subsequently increased mortality.^16^ The risk of spontaneous aneurysm rupture in pregnant women is 24% with a maternal and fetal mortality of 70–75% and 95%, respectively.^1,13,7,16,26,69^ Fortunately, recent advances in radiology and earlier diagnoses have lowered the rupture rate to below 3%. Pseudoaneurysms have a significantly higher rupture risk than true aneurysms (37%), which is related to the histopathological features of the aneurysm wall.^12,16,69^ Spontaneous SAA rupture often results in sudden pain in the upper-left quadrant, epigastrium, or left shoulder (due to diaphragmatic irritation), and hemodynamic instability related to hypovolemic shock.^4,12^ Pregnancy, clinically symptomatic aneurysm, diameter ≥2 cm, increase in diameter, surgical treatments influencing portal system pressure (e.g., portocaval shunt), portal hypertension, and liver transplantation are among the leading risk factors for rupture.^28^ Of the 78 giant SAA cases in this study, intraperitoneal or retroperitoneal rupture was diagnosed in 15 (19.2%) patients during preoperative radiological studies or intraoperative exploration. The mean aneurysm dimension in the cases with rupture was 100 ± 49.3 mm versus 96.8 ± 45.2 mm without rupture. In addition, 33.3% of the patients with intraperitoneal rupture died, and more than half of these were male (73.3%). These results suggest a tight correlation between aneurysm dimensions and rupture risk. Although previous studies highlight the tight correlation between pregnancy and rupture risk, and suggest that female patients are prone to rupture, our findings suggest that this should be reassessed.

Splenic artery aneurysms fistulize into the surrounding organs by destroying their walls and can present with gastrointestinal bleeding. Of SAA rupture cases, 13% are complicated with a colon, stomach, duodenum, or pancreatic duct fistula.^69^ Gastrointestinal fistulization of aneurysms can result in hematemesis, hematochezia, melena, and anemia. In our study, gastrosplenic artery fistulas were present in 3 patients^6,11,13^, a colo-splenic artery fistula was present in 1 patient^12^, and an SAA-pancreatic duct fistula was present in 1 case.^27^ Pancreatic duct involvement develops via either the direct destruction of the pancreatic duct or indirectly via a pancreatic pseudocyst.

Splenic artery aneurysms can result in arteriovenous fistulas by destroying the splenic vein or portal vein walls. In addition, they can lead to portal hypertension (predominantly left-sided), venous congestion, and venous collateral generation due to external pressure of the aneurysm on the splenic or portal vein. These complications are more frequent in giant SAAs.^33^ Our literature survey detected SAA-splenic vein fistulas in 3 cases and a SAA-portal vein fistula in 1 case.^17,25,39,47^ In addition, in cases with either a fistula or external pressure, various collateral veins developed.^22,47^

### Diagnosis

The tools used most frequently for diagnosing SAA are abdominal US, Doppler US, CT, magnetic resonance imaging, magnetic resonance angiography (MRA), endoscopic US, and digital subtraction angiography (DSA).^26,69^ US, an inexpensive, radiation-free diagnostic tool, is the first choice, especially for pregnant patients. However, obesity, gas artefacts, and a relatively lower sensitivity for smaller aneurysms are disadvantages of US.^13^ Multidetector CT, MRI, and MRA provide three-dimensional cross-sectional images. Although MRI and MRA are more sensitive and specific, their contraindication for patients with pacemakers and metal prostheses, potential emotional and respiratory problems for claustrophobic and respiratory distressed patients, relatively longer procedure duration, and unavailability on an emergency basis limit their use for diagnosis.^13^ Contrast-enhanced CT and CT angiography are quite helpful in the diagnosis of SAA; the typical aneurysm body is seen in the arterial phase.^16,22^ The major disadvantages of CT are the radiation dose, limited use in pregnant patients, and risk of contrast-nephropathy. However, multidetector CT is remarkably beneficial in differentiating SAAs from pancreatic tumors, pseudocysts, solid epithelial tumors, and gastric leiomyomas. Although DSA is the gold standard for diagnosis, the invasive nature of the procedure, involving arterial puncture and related complications, is a major disadvantage.^13,26^ The most important advantages of DSA are its ability to determine the exact location of the aneurysm, and the simultaneous detection and endovascular treatment of coexisting vascular abnormalities.^13,20^ In this study, 46.1% of the cases underwent diagnostic angiography, and 55.5% of these were treated simultaneously with the angiographic procedure.

### Management

Although there is no consensus on the management of SAA patients, there have been major changes as a result of progress in radiological diagnosis and treatment options.^1^ Regardless of their dimensions, all symptomatic SAAs are believed to require treatment.^1^ Treatment is also recommended for asymptomatic patients with lesions with dimensions ≥2 cm, who are pregnant or fertile, have portal hipertension, or are candidates for liver transplantation.^1,3,7,8,13,15^ The histopathological features of aneurysms also interfere with their evaluation. In true aneurysms, lesions with dimensions ≥2 cm have a significantly high risk of rupture and treatment is recommended. In comparison, the rupture risk for pseudoaneurysms is not related to their dimensions and all pseudoaneurysms should be treated.^5^

The treatment options for SAAs depend on age, sex, aneurysm dimension, location, complications, and severity of the clinical findings.^1,12,16,69^ The most frequent treatment options for SAAs are open abdominal surgery, endovascular treatment (coil embolization or stent), laparoscopic surgery, which is becoming more popular, and medical treatment.^1,8,69^ Despite technical improvements, open abdominal surgery remains the gold standard for treatment. Proximally located, elongated, and tortuous SAAs are suitable for *aneurysmectomy* and end-to-end reconstruction. This method preserves the spleen, an important element of the immune system.^13^ In contrast, a splenectomy might be added to the aneurysmectomy for lesions originating from the distal two-thirds of the splenic artery.^9,16^ For giant SAAs or cases where a simple aneurysmectomy is impossible due to dense strictures, preferable surgical options are an aneurysmectomy plus splenectomy, bipolar splenic artery ligation with/without aneurysmectomy, transaneurysmal splenic artery ligation, and distal pancreatectomy when necessary.^13,69^ The mortality and morbidity of open abdominal surgical treatment are 1.3% and 9%, respectively.^69^ Our literature survey revealed that surgical treatment often involved the spleen, pancreas, and other neighboring organs. This depended on the dimensions of the lesion, coexisting morbidities (pancreatitis, cirrhosis, or portal hipertension), and technical experience of the team. It is also necessary to address the infected (mycotic) SAA, usually secondary to infective endocarditis. Due to the high rupture risk for infected aneurysms, the best surgical option is aneurysmectomy plus splenectomy (in the presence of a splenic abscess and infarct).^37,49^

Endovascular treatment options are becoming more favored due to their acceptable technical success and low morbidity rates.^5,9,13^ The most frequent endovascular techniques are transcatheter embolization, percutaneous injection, and endovascular stent graft.^15^ Transcatheter embolization was initially introduced by Probost et al in 1978. Improvements in DSA technology and equipment allow embolization with success rates of 55–100%.^5,15^ Gelfoam gelatin, steel coils, detachable balloons, a detachable vascular plug, n-butyl cyanoacrylate glue, and thrombin are often used for embolization.^8,28^ Embolization is currently the first option for asymptomatic lesions diagnosed incidentally. Transcatheter embolization is preferred in cases involving surgical technical difficulty and in patients at increased operative risk. In addition, this option is considered for lesions located in the splenic hilum. The most frequent complications of transcatheter embolization are coil migration, aneurysm rupture, intestinal infarct, fever, splenic infarct, and abscess.^5,12,18^ In addition, an aneurysm might recanalize despite successful embolization. In such cases, reembolization or open abdominal surgical treatment might be preferred.^5^ The studies analyzed included a case report of coil migration into the stomach.^6^ Percutaneous injection is an option for cases in which transcatheter treatment is not suitable or has failed. This technique involves direct coil application or thrombin injection into the lesion.^15,45^ The most recent progress in SAA treatment is endovascular stenting.^69^ This minimally invasive technique preserves splenic perfusion via a stent placed in the aneurysm and excludes the dilated aneurysmatic segment. The stents used most frequently are self-expanding and balloon-expanding ones. Just as with embolization, tortuous arteries, decreased artery dimensions, and the location of the lesion limit application of this technique.^5^ The endovascular stent technique minimalizes splenic infarction and the abscess complications of coil embolization.^15^ Among the studies reviewed, there was only 1 case report involving endovascular stent graft application.^15^

A combination of several treatment techniques might be necessary for some cases, particularly for giant SAAs or patients with comorbid conditions. The initial embolization is followed by open abdominal or laparoscopic surgery. In our survey, open abdominal surgery was performed in three cases due to the failure of an embolization procedure.^6,18,35^

Laparoscopic SAA excision is a minimally invasive alternative to open abdominal surgery and was initially described by Saw et al in 1993.^69^ Despite its safety and applicability, this procedure requires experience and intraoperative US. It is contraindicated in hemodynamically unstable patients or those at rupture risk. Laparoscopic excision can be the optimal treatment, particularly in early pregnancy and with small lesions.^7^ However, it is not suitable for larger aneurysms and lesions with dense adhesions to surrounding tissues. Most importantly, patient safety requires technical experience. There was no laparoscopic surgery in our survey.
